# Dynamic Power-Conscious Routing for MANETs: An Initial Approach

**DOI:** 10.6028/jres.104.037

**Published:** 1999-12-01

**Authors:** Madhavi W. Subbarao

**Affiliations:** National Institute of Standards and Technology, Gaithersburg, MD 20899-0001

**Keywords:** decentralized networks, dynamic routing, mobile ad hoc networks, power efficiency

## Abstract

We develop an initial dynamic power-concious routing scheme (MPR) that incorporates *physical* layer and *link* layer statistics to conserve power, while compensating for the channel conditions and interference environment at the intended receiver. The aim of MPR is to route a packet on a path that will require the least amount of total power expended and for each node to transmit with just enough power to ensure reliable communication. We evaluate the performance of MPR and present our preliminary results.

## 1. Introduction

A mobile ad hoc network (MANET) is an autonomous collection of mobile nodes that communicate over relatively bandwidth-constrained wireless links. Significant examples of MANETs include establishing survivable, dynamic communication for emergency/rescue operations, disaster relief efforts, and military networks. MANETs need efficient distributed algorithms to determine network organization (connectivity), link scheduling, and routing. Message routing in a decentralized environment where network topology fluctuates is not a well-defined problem. Factors such as variable wireless link quality, propagation path loss, fading, multiuser interference, and topological changes, become relevant issues.

In addition to the characteristics mentioned, an important issue in network routing for MANETs is to conserve power while still achieving a high packet success rate. This can be accomplished by altering the transmitter power to use just that amount needed to maintain an acceptable signal-to-noise ratio (SNR) at the receiver. Reducing the transmitter power allows spatial reuse of the channel and thus, increases network throughput [1]. Altering the transmission power also reduces the amount of interference caused to other networks operating on adjacent radio frequency channels. In networks where nodes operate on battery power, conserving power is crucial since battery life determines whether a network is operational or not. Military networks desire to maintain a *low probability of intercept* and/or a *low probability of detection* [2]. Hence, nodes prefer to radiate as little power as necessary and transmit as infrequently as possible, thus decreasing the probability of detection (or interception).

The benefits of power conservation/control for MANETs prompt the important question: What is the most power efficient way to route a packet from a source to a destination such that the packet is received with an acceptable packet success rate [3]? Since channel conditions and multiuser interference levels are constantly changing with time, the transmitter power necessary on a particular link must be determined dynamically. In Ref. [4], Wieselthier, Nguyen, and Ephremides address this problem in the context of wireless multicasting, and in Ref. [5], Pursley, Russell, and Wysocarski consider this problem in a frequency-hopping ad-hoc network.

In this paper, we conduct an initial investigation on the effects of energy-efficient wireless routing in MANETs. We develop an initial dynamic power-conscious routing scheme (minimum power routing or MPR) that incorporates physical layer and link layer statistics to conserve power, while compensating for the propagation path loss, shadowing and fading effects, and interference environment at the intended receiver. The main idea of MPR is to select the path between a given source and destination that will require the least amount of total power expended, while still maintaining an acceptable SNR at each receiver. A “cost” function is assigned to every link reflecting the transmitter power required to *reliably* communicate on that link. As an initial approach, the distributed Bellman-Ford algorithm can be used to perform “shortest” path routing with the cost functions as the link distances. The resulting “shortest path” is the MPR path from a given source to a destination. We compare the performance of MPR to that of shortest distance routing with power control (SD-PC) and minimum hop routing with power control (MH-PC), and present our preliminary results.

## 2. Power-Concious Routing

### 2.1 System Model

Consider a transmitter communicating with a receiver at a distance of *r*_0_ in a MANET. As the transmitted signal propagates to the receiver, it is subject to the effects of shadowing and multipath fading, and its power decays with distance, i.e., $P_{R}\propto KFP_{T}r_{0}^{-\eta }$, where *K* is a constant, *F* is a non-negative random attenuation for the effects of shadowing and fading, *P*_T_ is the transmitter power, and *η* is the path loss exponent. At the receiver, the desired signal is corrupted by interference from other active nodes in the network. We assume that nodes know the identity of all other nodes in the network and the distances to their immediate neighbors, i.e., nodes that are within transmission range. Interfering nodes use the same modulation scheme as the transmitter and nodes can vary their transmit power up to a maximum power *P*_max_. We assume that the multiuser interference is a Gaussian random process. At the receiver, the decoder maintains an estimate of the average SNR.

### 2.2 Minimum Power Routing Protocol

The aim of MPR is to route a packet on a path that will require the least amount of total power expended and for each node to transmit with just enough power to ensure that the transmission is received with an acceptable bit error rate *ϒ*. Threshold *ϒ* is a design parameter and may be selected according to the network performance desired. Let ***ε*** be the bit-energy-to-noise-density ratio, $\varepsilon_{b}/N_{0_{\text{eff}}}$, necessary at a node to achieve *ϒ*.

Without loss of generality, consider a transmission from node *i* to node *j*, where *i* ≠ *j*, and *i*, *j* ∈ {1,…, *N*}, where *N* is the number of nodes in the network. The received $\varepsilon_{b}/N_{0_{\text{eff}}}$ is given by

$${\left[\frac{\varepsilon_{b}}{N_{0_{\text{eff}}}}\right]}_{ij}=\frac{P_{R_{ij}}/D}{N_{0}+P_{I_{ij}}/W},$$ (1)

where *D* is the data rate in bits per second, *W* is the system bandwidth in hertz, $N_{0}/2$ is the power spectral density of the thermal noise, $P_{I_{ij}}$ is the power of the interference at node *j* due to all nodes excluding node *i*, and $P_{R_{ij}}$ is the received power at node *j* due to node *i*. From the description in Sec. 2.1, it follows that the received power is given by

$$P_{R_{ij}}=KF_{ij}P_{T_{ij}}r_{ij}^{-\eta },$$ (2)

where $P_{T_{ij}}$ is the transmitter power used at node *i* to communicate with node *j*, *F_ij_* is a non-negative random attenuation for the effects of shadowing and fading on link *ij*, and *r_ij_* is the distance between node *i* and node *j*. Substituting Eq. (2) into Eq. (1), we obtain

$${\left[\frac{\varepsilon_{b}}{N_{0_{\text{eff}}}}\right]}_{ij}=S_{ij}P_{T_{ij}}r_{ij}^{-\eta },$$ (3)

where

$$S_{ij}\frac{KF_{ij}}{D\left(N_{0}+P_{I_{ij}}/W\right)},$$ (4)

may be interpreted as a dynamic *link* scale factor reflecting the current channel characteristics and interference on link *ij*. These scale factors reflect a link’s most recent reception environment. Note that *S_ij_* ≠ *S_ji_* since channel conditions are not symmetric.

It is desirable for ${\left[\varepsilon_{b}/N_{0_{\text{eff}}}\right]}_{ij}$ to equal the energy ratio ***ε***, since this is the minimum $\varepsilon_{b}/N_{0_{\text{eff}}}$ necessary to achieve the bit error rate *ϒ*. Hence, with knowledge of scale factor *S_ij_*, node *i* can easily determine the power $P_{T_{ij}}$ necessary to achieve this goal using Eq. (3), i.e.,

$$P_{T_{ij}}=\frac{\varepsilon }{S_{ij}r_{ij}^{-\eta }}.$$ (5)

Let ${\left[\varepsilon_{b}/{\hat{N}}_{0_{\text{eff}}}\right]}_{ij}$ be an estimate of the received bit energy ratio at the output of the decoder at node *j*. Many methods may be used to determine ${\left[\varepsilon_{b}/{\hat{N}}_{0_{\text{eff}}}\right]}_{ij}$, e.g., using side information by embedding known test symbols in packet transmissions [6]. Although $P_{T_{ij}}$ was selected to achieve energy ratio ***ε*** at the receiver, since network conditions are changing, the actual received ${\left[\varepsilon_{b}/N_{0_{\text{eff}}}\right]}_{ij}$ may differ from ***ε***. If node *j* has knowledge of the transmitter power $P_{T_{ij}}$ (which can be accomplished by including $P_{T_{ij}}$ in the packet header), it can update its estimated scale factor using a smoothing function as follows,

$${\hat{S}}_{ij}=(1-\alpha )\cdot \frac{{\left[\varepsilon_{b}/{\hat{N}}_{0_{\text{eff}}}\right]}_{ij}}{P_{T_{ij}}r_{ij}^{-\eta }}+\alpha \cdot {\hat{S}}_{ij},$$ (6)

which mitigates the fluctuations due to multiuser interference (and *α* is a smoothing factor). An initial value for *Ŝ_ij_* may be computed as described in Sec. 2.3. The estimated link scale factor *Ŝ_ij_* accounts for variable channel conditions and for all types of Gaussian interference, e.g., multiuser interference and partial-band jamming. If the received bit error rate *ϒ_ij_* on link *ij* is less than threshold *ϒ*, the effect of Eq. (6) is that node *j* decreases its link *Ŝ_ij_* value, indicating an *increase* in its interference (noisy channel) level, and thus, an *increase* in the power necessary to communicate on link *ij* as computed by Eq. (5). The opposite behavior occurs when *ϒ_ij_* is greater than *ϒ*.

Each time node *j* receives a packet from a node *i*, it computes and stores a value for *Ŝ_ij_* that accurately reflects its current SNR on link *ij*. We assume that the rate of change of the network is much slower than a packet transmission interval, and hence the value for *Ŝ_ij_* is valid for many packet transmissions.

For every pair of nodes *i* and *j*, a cost *C_ij_* given by

$$C_{ij}=\{\begin{array}{ll} P_{T_{ij}}\left(1+\kappa \right)\; & \text{if}\;P_{T_{ij}}\left(1+\kappa \right)\leq P_{\max},\\ \infty & \text{otherwise}, \end{array}$$ (7)

is assigned, where *κ* is a dampening constant to inhibit oscillations. The inequality in Eq. (7) is necessary since the transmitter power is limited by *P*_max_. The cost *C_ij_* is the power necessary to communicate from node *i* to node *j* to compensate for channel conditions and interference. Since nodes only know *estimates* of the link scale factors, the power required on a link must be overplayed. Thus, *κ* provides an extra margin for the transmission power and is a design parameter that must be selected. As an initial approach, the distributed Bellman-Ford algorithm can be used to perform “shortest” path routing with the *C_ij_* as the link distances. The resulting “shortest path” is the MPR path from a given source to a destination. If there is more than one path with the same minimum total cost, the MPR path is chosen as the one with the smallest maximum cost on any one link. MPR avoids congested areas and is also *minimax* optimal, i.e., given some uncertainty in the link scale factors, it minimizes the worse case total path cost.

### 2.3 Network Implementation

Initially, nodes transmit using power *P*_max_, and the cost of every link is set to a constant *d*, where *d* = *P*_max_(1 + *κ*). This will result in nodes initially routing packets according to the *minimum number of hops* to the destination. The first time node *j* for *j* ∈ {1,…, *N*}, receives a transmission from another node, say node *i*, it will compute its link scale factor *Ŝ_ij_*, i.e,

$${\hat{S}}_{ij}=\frac{{\left[\varepsilon_{b}/{\hat{N}}_{0_{\text{eff}}}\right]}_{ij}}{P_{\max}r_{ij}^{-\eta }}.$$ (8)

The link costs will be computed as described in Sec. 2.2 and propagated throughout the network. If the cost of a particular link has not yet been computed within a specified amount of time because no data packet was transmitted on that link, a “boost” packet is transmitted on the link and the link cost is computed. Once all of the link costs have been computed, the routing protocol is now MPR.

The MPR path costs must be periodically circulated around the network. This information can be passed around via data packets, acknowledgments, and special control packets known as packet radio organization packets (PROPs) [7]. For this initial implementation, we assume an underlying information dissemination scheme.

A dynamic routing table is maintained by each node. For each destination, a node stores the outgoing link for the most power-efficient route and the corresponding path cost, distance to the destination, and the necessary transmitter power. Since network conditions are changing, routing tables are continually updated based on an *update interval*, and the transmission power is altered on a per packet basis according to Eq. (5). Before an update, if a link cost is deemed *out-dated*, i.e., the cost has not been recomputed within a specified interval before an update, a “boost” packet is transmitted on that link in order to compute a current link cost.

## 3. Performance of Power Conscious Routing

We compare the performance of MPR to that of SD-PC and MH-PC, and present our preliminary results. The transmission power for SD-PC and MH-PC is altered to overcome the distance between the transmitter and intended receiver. We use the modeling and simulation tool OPNET to build a network prototype and execute the simulations. We assume a MANET using the ALOHA random access protocol. We consider a slow fading (log-normal shadowing) environment, and vary the random attenuation effects on a link every *T*_S_ seconds according to a *β* correlation factor. We assume that a node has knowledge of the transmitter power used to communicate with it and hence, uses Eq. (6) to update the estimate of its link scale factor. A list of the simulation parameters is given in Table 1.

Performance measures of *end-to-end throughput*, *end-to-end delay*, *efficiency*, and a*verage power expended* are used to analyze the performance of the routing protocols. End-to-end throughput is defined as the number of packets that successfully reach their final destination per unit time. End-to-end delay is based only on successful packets and is defined as the average time required for a packet to arrive at its destination. Efficiency is the number of received data packets divided by the total number of data packets and control packets transmitted. Average power expended is the average power consumed in the network relaying successful packets (including necessary control packets) from their source to their final destination per unit time.

First, we consider a 16 node static network with packet generation rate per node *ρ* = 10 s^−1^ and a total of 10000 packets being exchanged. The routing table update interval is 10 s, and the shadowing parameters are *β* = 0.8 and *T*_S_ = 5 s. From Table 2, we see that MPR achieves approximately *double* the throughput for similar power consumption levels, or alternatively, requires approximately 2.5 times *less* power for similar throughput levels. The overall end-to-end delay is comparable for all schemes. While MPR does not optimize on the number of hops, it routes around undesirable links and hence, requires overall lower power consumption.

**Fig. 1:**
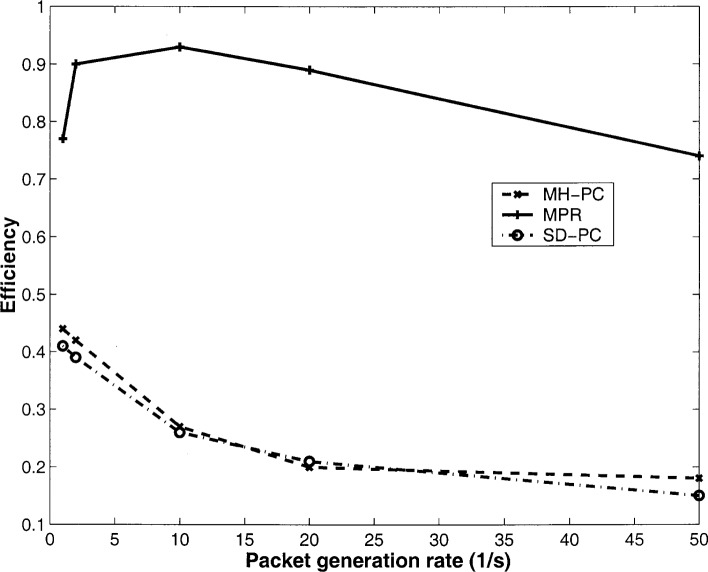
Efficiency vs packet generation rate *ρ*.

**Fig. 2:**
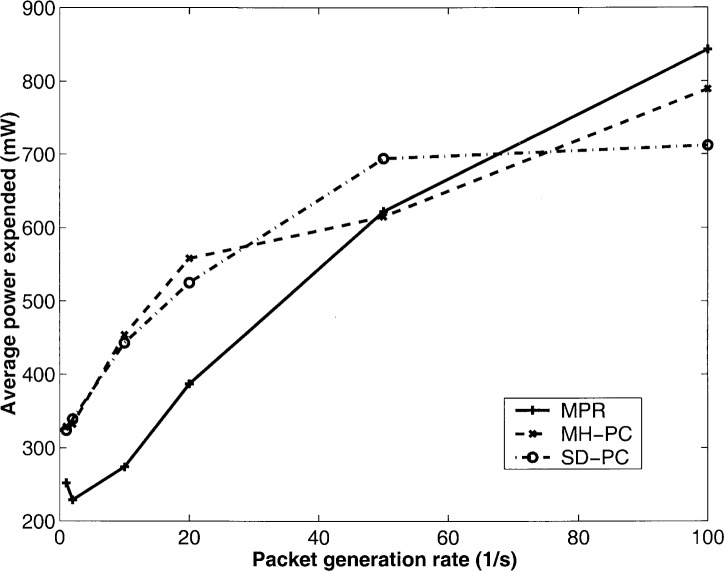
Average power expended vs packet generation rate *ρ*.

Next, with the same network configuration, we vary the packet generation *ρ* and plot the efficiency and average power expended in Figs. 1 and 2, respectively. We see that as *ρ* increases, the efficiency increases until the point where further packet generation causes excess levels of network traffic, and thus, a decrease in efficiency. MPR achieves approximately double the efficiency as SD-PC and MH-PC for low values of *ρ* and approximately a striking 4.5 times higher efficiency for larger values of *ρ*, since MPR adapts to changing interference levels. For low values of *ρ*, MPR utilizes from 30 % to 50 % less power relaying successful packets than SD-PC and MH-PC. For higher values of *ρ*, although MPR utilizes approximately 50 mW more power than SD-PC and MH-PC, since both MH-PC and SD-PC achieve low efficiency, most of the total power expended in those schemes is on unsuccessful transmissions.

**Fig. 3:**
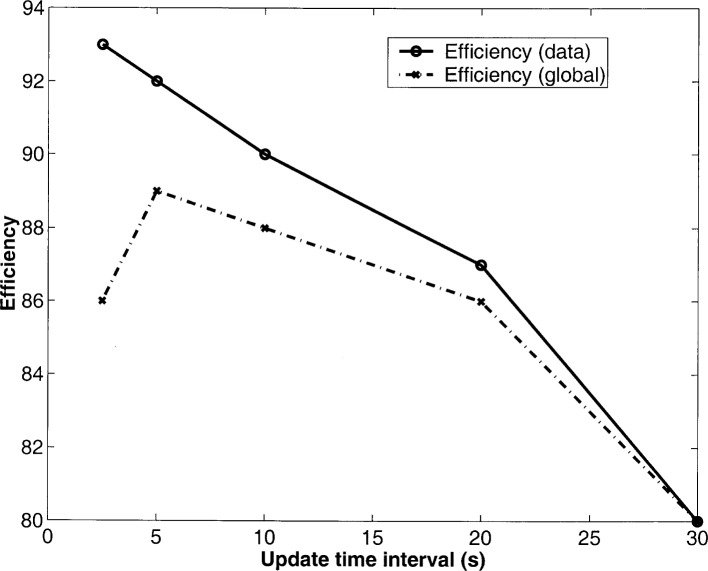
MPR: efficiency vs update time interval (s).

Finally, we introduce mobility into the network with nodes moving at a speed of 4 m/s and investigate the effect of different routing table update intervals on MPR. The packet generation rate per node is *ρ* = 10 s^−1^. In Fig. 3, we plot the network efficiency verses update time interval (s). We consider the efficiency of only data transmissions, and the global efficiency of both data and control packets, i.e., data packets received divided by total communication packets—both data and control. We see that as the update interval decreases, the data efficiency increases since the routing information utilized is more current. However, the global efficiency increases until it reaches a point where further updates cause too much overhead communication, and hence, a decrease in network efficiency. Clearly, there is a trade-off between utilizing current routing information and the communication overhead generated. It is our conjecture, that the optimum update interval is the same as the slow fading duration *T*_S_.

## 4. Conclusion

We conducted an initial investigation of energy-efficient wireless routing in MANETs. We presented our preliminary results and conclude that MPR shows promise as a power conscious routing scheme for MANETs. MPR adapts to the changing channel conditions and interference environment of a node. The power-conscious concepts developed herein can be adopted in other MANET routing algorithms.

## Figures and Tables

**Table 1:** Network simulation parameters

Parameter	Value
Network area	900 m × 600 m
Data rate	1 Mbit/s
Max TX power/range	500 mW/250 m
Min frequency	2.4 GHz
Bandwidth	83 MHa
Modulation	Direct-Sequence BPSK
Processing gain	20 dB
Packet length	100 bit
Shadowing	10 log *F* ≈ *N*(0, 64 dB^2^)
*ϒ*, *η*, *α*, *κ*	3 × 10^−4^, 2.6, 0.8, 0.2

**Table 2:** Simulation results for a 16 node static network

Measure	MPR	SD-PC	SD-PC	MH-PC	MH-PC
Hops	30682	24945	15321	25075	17485
Overhead	0.0077	0	0	0	0
Pk delay^a^ (μs)	28.5	24.5	26	24.8	27.6
Pk pwr^a^ (mW)	305	660	279	702	266
Hop pwr^a^ (mW)	91.3	244	94.1	255	91.3
Efficiency	0.95	0.92	0.51	0.92	0.6
Packet throughput (s^−1^)	9.58	9.2	5.15	91.3	5.7

a Mean value of three trials.
